# Assessment of the Anti-Protozoal Activity of Crude *Carica papaya* Seed Extract against *Trypanosoma cruzi*

**DOI:** 10.3390/molecules181012621

**Published:** 2013-10-11

**Authors:** Matilde Jiménez-Coello, Eugenia Guzman-Marín, Antonio Ortega-Pacheco, Salud Perez-Gutiérrez, Karla Y. Acosta-Viana

**Affiliations:** 1 Laboratorio de Biología Celular, CIR “Dr. Hideyo Noguchi”, CA Biomedicina de Enfermedades Infecciosas y Parasitarias, Universidad Autónoma de Yucatán, Ave. Itzaes # 490 x 59, Centro C. P. 97000, Mérida Yucatán, Mexico; E-Mails: mjcoello@uady.mx (M.J.-C.); gmarin@uady.mx (E.G.-M.); 2 Departamento de Salud Animal y Medicina Preventiva, CA Salud Animal, Facultad de Medicina Veterinaria y Zootecnia, Universidad Autónoma de Yucatán, Km. 15.5 Carr. Merida-Xmatkuil AP. 4-116 Mérida, Yucatán, Mexico; E-Mail: opacheco@uady.mx; 3 Universidad Autónoma Metropolitana-Xochimilco, Calzada del Hueso # 1100. C.P. 04960, México D.F. A.P. 23–181, Mexico; E-Mail: msperez@correo.xoc.uam.mx

**Keywords:** Chagas’ disease, antiprotozoal, fatty acids, *in vivo*, *Carica papaya*

## Abstract

In order to determine the *in vivo* activity against the protozoan *Trypanosoma cruzi*, two doses (50 and 75 mg/kg) of a chloroform extract of *Carica papaya* seeds were evaluated compared with a control group of allopurinol. The activity of a mixture of the three main compounds (oleic, palmitic and stearic acids in a proportion of 45.9% of oleic acid, 24.1% of palmitic and 8.52% of stearic acid previously identified in the crude extract of *C. papaya* was evaluated at doses of 100, 200 and 300 mg/kg. Both doses of the extracts were orally administered for 28 days. A significant reduction (*p* < 0.05) in the number of blood trypomastigotes was observed in animals treated with the evaluated doses of the *C. papaya* extract in comparison with the positive control group (allopurinol 8.5 mg/kg). Parasitemia in animals treated with the fatty acids mixture was also significantly reduced (*p* < 0.05), compared to negative control animals. These results demonstrate that the fatty acids identified in the seed extracts of *C. papaya* (from ripe fruit) are able to reduce the number of parasites from both parasite stages, blood trypomastigote and amastigote (intracellular stage).

## 1. Introduction

Chagas’ disease is an important neglected public health problem in many Latin American countries [1] which is caused by an infection with the intracellular protozoan parasite *Trypanosoma cruzi* [2]. The chemotherapy for Chagas disease is not satisfactory due to the partial effectiveness and the toxicity associated with the available long-term treatments [3]. The most common drugs used to treat the disease are nifurtimox (3-methyl-4(5-nitrofurfurylideneamino)-tetrahydro-[1,4]thiazine-1,1-dioxide) and benznidazole (*N*-benzyl-2-nitro-1-imidazoleacetamide), both developed over four decades ago, which represent the sole chemotherapeutic options for treating this highly neglected disease. Beside, both drugs present variable efficacy depending on the geographical area and the occurrence of natural resistance, and show low antiprotozoal activity against the later chronic stage [2]. The treatment is urgently indicated during the acute phase and during the reactivation of the disease (especially during immunosuppression) [4] but there are no available resources for treatment for the chronic phase of disease and neither is there any available vaccine.

Research and development of new drugs effective for the treatment of *T. cruzi* infection is a real and pressing need and requires new strategies for drug development, to supply infected patients alternatives for chemotherapy without causing adverse reactions associated with toxicity. Natural products from e traditional Mexican medicine have provided molecules with drug-like properties and high structural diversity [5,6].

*Carica papaya* (Linn), commonly known as papaya, is a fruit crop cultivated in tropical and subtropical regions, and well known for its nutritional benefits and medicinal applications [7]. It is widely distributed in the south of Mexico including the Caribbean and grows at an altitude range of 10 to 1,600 m above sea level. The antimicrobial, antifungal, larvicidal [8] and antiprotozoal properties of *C. papaya* against *Trichomona vaginalis* have been previously reported [9].

The present study evaluated the *in vivo* antiprotozoal activity of crude *C.* *papaya* seeds extract and its main components against *T. cruzi* infective forms (blood trypomastigotes and amastigotes), during the acute phase of the disease.

## 2. Results

The evaluation of chloroform extract of the seeds of *C. papaya* during the acute phase with doses of 50 mg/kg, 75 mg/kg and 100 mg/kg of a mixture of the major fatty acid components (stearic acid, palmitic acid and oleic acid) showed antiprotozoal activity against trypomastigotes of *T. cruzi*, however total elimination of the parasites was not observed with any of the doses tested.

**Figure 1:**
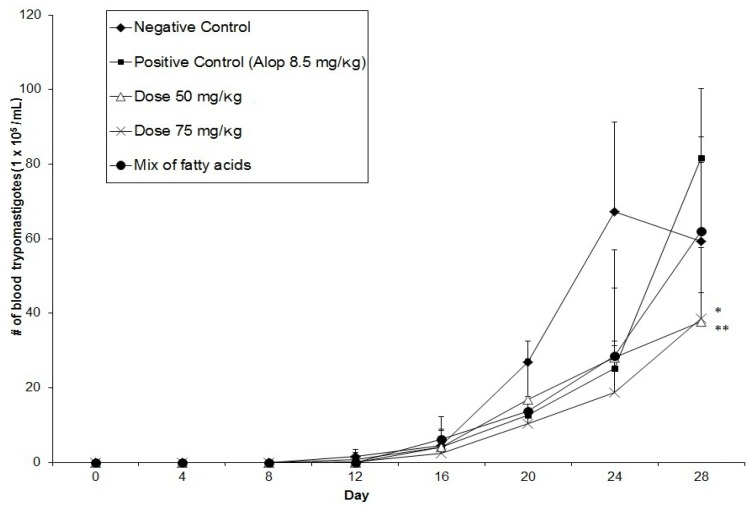
Effect of chloroform extract of *Carica papaya* on the growth curve of trypomastigotes of *T. cruzi* of BALB/c mice during the acute phase of infection (* and ** *p* *<* 0.05).

With the crude chloroform extract at doses of 50 and 75 mg/kg, a reduction in the number of parasites of 54 and 54% respectively was observed compared with the positive control (allopurinol 8.5 mg/kg) and animals treated with the mixture of compounds showed a 40% reduction when were compared with the positive control group. The 50 and 75 mg/kg doses also exhibited an antiprotozoal activity increase throughout the experiment to be below the negative control group (35 and 34% respectively) (Figure 1).

Although an antiprotozoal effect on parasitemia levels was observed with *C. papaya* chloroform extract, the number of quantified amastigote nests in heart tissue of treated mice was not different from the negative control group. 

**Figure 2:**
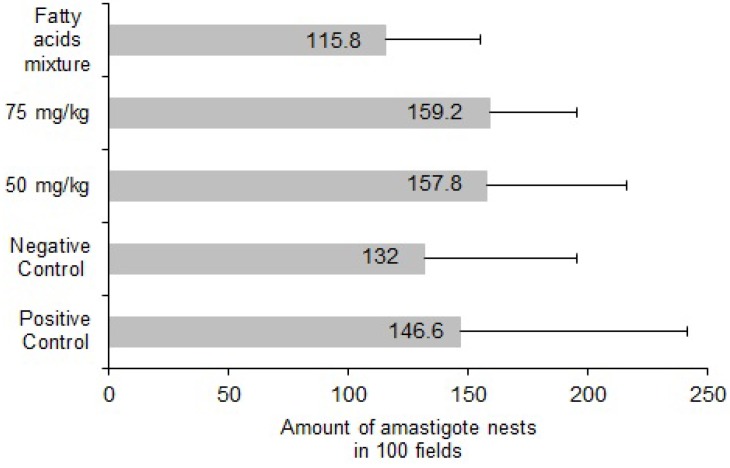
Effect of chloroform extract of seeds from *C. papaya* over the number amastigote nests observed in cardiac tissue from mice BALB/c infected with trypomastigotes of *T. cruzi* and treated at doses 50 and 75 mg/kg of chloroform crude extract and 100 mg/kg of the mixture of the fatty acid identified in the crude extract.

In contrast there was a slight reduction in the number of amastigote nests in cardiac tissue in the group treated with the mixture of fatty acids, and conversely the group of positive control animals (treated with allopurinol) demonstrated the greatest number of amastigote nests (Figure 2). 

**Figure 3:**
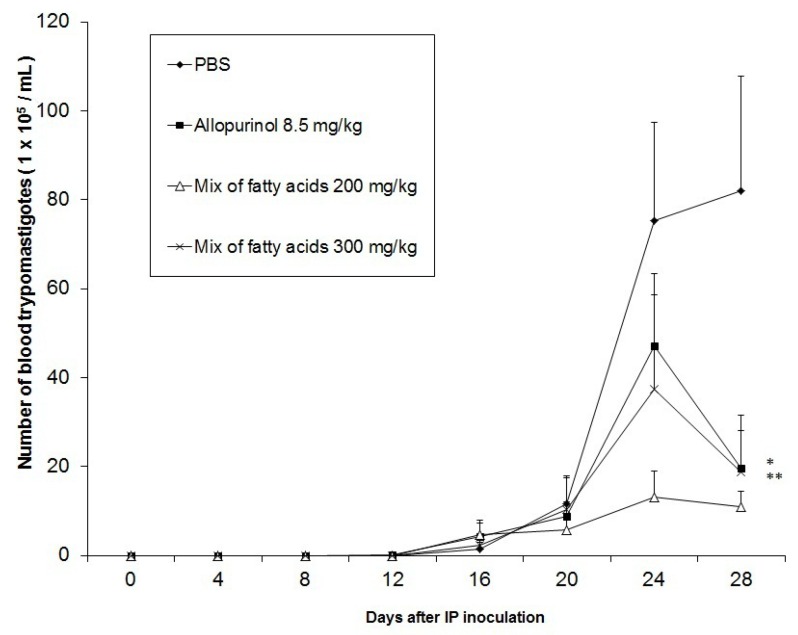
Effect of the mix of fatty acids identified in the chloroformic *Carica papaya* crude extract on the growth curve of trypomastigotes of *T. cruzi* (* and ** *p* < 0.05).

The results derived from monitoring of the doses of 200, and 300 mg/kg of the mixture which contained the three main compounds from the extract of *C. papaya* seeds (fatty acids) confirmed the antiprotozoal activity of these substances. In the group of animals treated with a 200 mg/kg dose a significantly lower parasite replication (*p* < 0.05) was observed, projecting an 84.5% reduction in trypomastigotes compared to negative control at 28 days post inoculation (Figure 3). 

Animals treated with 300 mg/kg reduced the amount of parasites by 77.73% compared with the negative control group.

**Figure 4:**
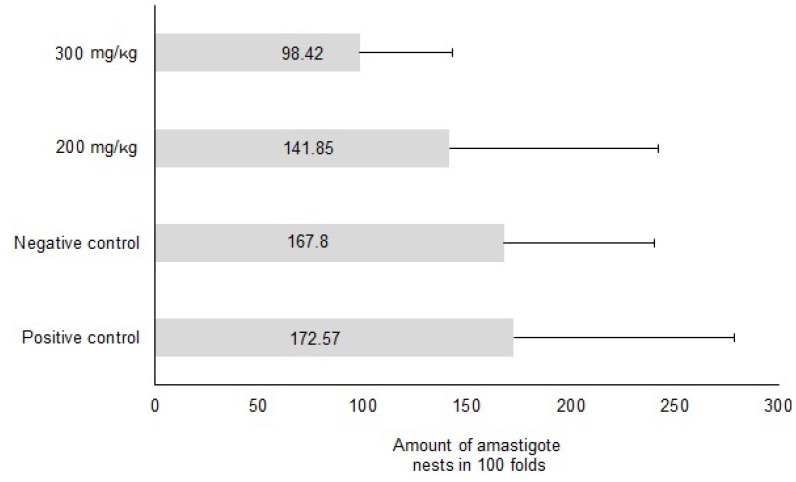
Effect of the mix of the fatty acids identified in the chloroformic crude extract of *C. papaya* seeds over the number amastigote nests observed in cardiac tissue from mice BALB/c infected with trypomastigotes of *T. cruzi* and treated at doses 200 and 300 mg/kg.

When the antiprotozoal activity of the mixture of the main compounds of the *C. papaya* chloroform extract was evaluated with two different doses, it was noted that the greatest reduction in the number of amastigote nests was seen in animals treated with 300 mg/kg of the mixture of fatty acids identified in the chloroform extract of seeds of *C. papaya* (43%) compared to positive control animals. Likewise, animals treated with the mixture of fatty acids but with a 200 mg/kg dose showed a slight decrease in the number of nests (18%) compared to the positive control group (allopurinol); those two had the highest number of amastigote nests (x = 172.57, SD. 106.4) (Figure 4).

In the first bioassay evaluating two different chloroform extract doses and the single dose of 100 mg/kg of the mixture of fatty acids, a higher mortality in the positive control animals group (57%) was registered, followed by the group treated with the fatty acids mixture at a 100 mg/kg dose (28%). There was also lower mortality in animals treated with the *C. papaya* chloroform extract, where in the animals treated with a dose of 50 mg/Kg no mortality was observed (*p* < 0.05 compared with both control groups) and the group treated with the 75 mg/kg dose recorded a 28.5% mortality rate.

**Figure 5:**
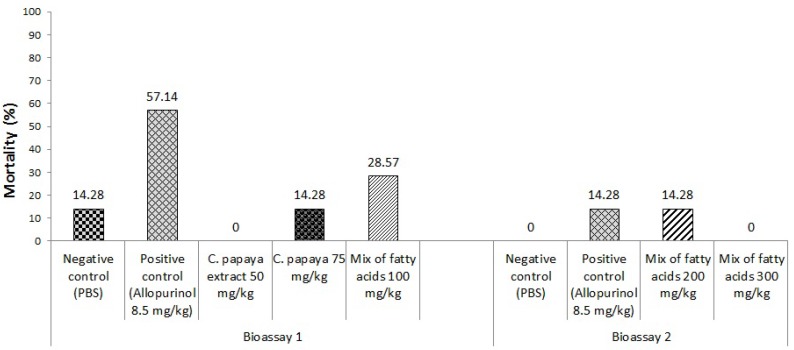
Effect of *C. papaya* extract (at doses of 50 and 75 µg/g) and the mix of fatty acids identified in the crude extract (at doses of 100, 200 and 300) on the mortality rate of BALB/c mice infected with *T. cruzi* and treated during 28 days.

Moreover, in the second bioassay, increased mortality was recorded in the positive control groups and the animals treated with the mixture of fatty acids at doses of 200 mg/kg (14.28%). In animals from negative control groups and treated with the fatty acids mixture at a 300 mg/kg dose there was no mortality (Figure 5).

**Figure 6:**
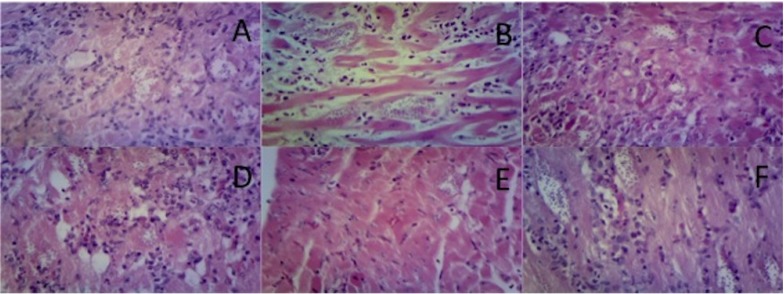
Microphotographs of the cardiac tissue of BALB/c (40×, HE). Untreated individuals (Negative Control) (**A**), treated with allopurinol (Positive Control, 8.5 mg/kg) (**B**), treated with seed CE of *C. papaya* (50 mg/kg) (**C**), treated with seed CE of *C. papaya* (75 mg/kg) (**D**), treated with the combination of FA (200 mg/kg) (**E**) and treated with the combination of FA (300 mg/kg) (**F**). [CE = chloroform extract, FA = Fatty Acids].

During the examination of the histological sections from the heart, in the negative control animals a significant amount of infiltrate (histiocytes), cardiac fiber breakage and a diffuse necrotic myocarditis were found (Figure 6A). In the positive control group, animals showed necrosis, inflammation of plasma cells and macrophages, as well as degenerate fibers and increased intercellular space caused by severe edema (Figure 6B). The tissue of animals treated with 50 mg/kg dose of *C. papaya*, a severe necrosis was observed, but lower than in the control groups (Figure 6C). Animals treated with a 75 mg/kg dose showed the presence of inflammatory infiltrate and degenerated heart muscle fiber damage (Figure 6A). Moreover, in animals treated with the mixture of fatty acids, it was observed that the 200 mg/kg dose still showed necrosis and numerous macrophages. but had no plasma cells (Figure 6E). In the animals which were administered the fatty acid mixture at 300 mg/kg dose, there were macro cell necrosis lymphocytes, slight inflammation and amastigote nests that were visibly smaller (Figure 6F).

## 3. Discussion

The results found in this study demonstrated a significant anti protozoan activity of *C. papaya* in mice treated with extracts as well in mice treated with the evaluated doses of the compound mixtures. These findings are consistent with other studies which described the *in vitro* activity of *C. papaya* against protozoan *Trichomonas vaginalis* at a 5.6 ug/mL concentration [9], and also had activity against the protozoa described in fish *Ichthyophthirius multifiliis* at a 200 mg/L concentration [10]. However these studies show that the antiprotozoal activity was observed for the trypomastigote forms, and the treatment with crude extract or compound mixture did not show any significant activity against the intracellular form of the parasite (amastigote). 

There are some other studies reporting that ground seeds and crude extract obtained from seeds of *C. papaya* showed significant activity on gastrointestinal parasites in humans [11]. The *in vitro* activity of aqueous and ethanol extracts of *C. papaya* seeds against eggs of the parasite *Heligmosomoides bakeri* (obtained from *Mus musculus*) at a 2.75 mg/mL concentration have also been reported [8].

The composition of the chloroform seed extract of *C. papaya* used in this research was previously determined by GC-MS and the oleic (45.97%), palmitic (24.1%) and stearic (8.52%) acids were the main components [12]. Recent studies have shown the antiprotozoal activity of derivatives of fatty acids against *Leishmania infantum* and *Leishmania major*; the fatty acids with medium chain derivatives (TyC8, TyC10 and TyC12) exhibit good antimicrobial and antileishmanial activities [13].

For these reasons, there is an increased interest on topics related to the activity of fatty acids and the synthesis of tyrosil-esters. Few data regarding the biological activities of tyrosyl esters has been reported, as well as antithrombotic agents have reduced the viability of some types of cancer cells. 

The results observed in this study showed a reduction in the number of blood trypomastigotes (*p* < 0.05) as well as amastigote nests; therefore a reduction in the mortality rate in treated animals compared to negative control animals was found. It is worth mentioning that drugs against *T. cruzi* tend to be highly toxic and relatively effective; the most commonly used drugs have been benznidazole and nifurtimox, nowadays nifurtimox currently is no longer produced and in Mexico it is not possible achieve any of these two drugs. For this reason is justified the identification of novel targets for chemotherapy including: inhibitors of sterol biosynthesis, inhibitors or substrates for purine salvage pathway, cysteine protease, and pyrophosphate metabolism among others [14], but all of them are also characterized complications of toxicity with their use. 

On the other hand, relating to *Plasmodium falciparum*, it has been described that for the oleic acid synthesis in this parasite, the catalytic activity of the enzyme steroayl-CoA desaturase (SCD) is necessary, beginning from the stearic acid, adding NADPH [15]. However, in this study when *T. cruzi* is exposed to high oleic acid concentrations, the product itself in excess could produce an alosteric inhibition to the SCD and with that affect the lipidic metabolic processes in the parasites. SCD is an iron-dependent enzyme which catalizes the insertion of a *cis* double bond at the Δ9 position of fatty acyl-CoAs with NADPH as a cofactor [16].

An advantage of the oral administration of chloroform extracts of *C. papaya* seeds, such as fatty acids, is that they do not seem to cause toxic effects, since it has been shown that can be safely used up to a 4,000 mg/kg dose in mice [12]; it has also been administered in Langur monkeys and domestic dogs for periods longer than 90 days without showing toxicological alterations during the time that was given (about 100 days or so) [17,18].

However, it must be considered that the chloroform extract made of *C. papaya* has a liquid consistency, and because it reaches a concentration around 1 μg/μL, in mice model is not feasible to evaluate higher doses. In other animal models it may be evaluated a better antiprotozoal activity at higher doses in search of a new alternative for the treatment against *T. cruzi*.

On the other hand, Ghosh *et al.* [19] described the utility of a Streptomyces-derived lipidated peptide metabolite, able to inhibit the growth of *Plasmodium falciparum*; the lipo-peptid had little or no activity against a panel of mammalian, fungal, and Gram positive bacteria cell lines. Those authors also mentioned that the novel lipoprotein main structure might provide interesting insights and differences in microbial and mammalian cell biology. 

The amastigote stage of *T. cruzi* is the only replicative stadium of the parasite in the mammalian host; this is an intracellular stadium and depending on the strain involved, tissue tropism is predominantly toward heart and muscle. The highly lipid solubility of the main components of the *C. papaya* chloroform extract allows an increased capacity of penetration trough the membrane cells. Presumably due to this good penetration, a 43% reduction in the number of amastigote nests in treated animals (200 mg/kg of the mixture of fatty acids) was recorded (Figure 4). Their use at higher doses or in combination with some other anti-chagasic drug might be a good option for the treatment of the disease, for which there are not many alternatives of treatment [14].

From the recently studied drugs for the treatment of Chagas disease, the most promising compounds are cruzipaine inhibitors and the inhibitors of ergosterol biosynthesis [20]. On the other hand, the liposomal amphotericin B has been described as being efficient to prevent mortality and reduces significantly parasitic loads in most tissues from a mouse model during the acute and chronic stages of infection [21].

There are reports describing that crude extracts from other seeds (*i.e.*, from the Cucurbitaceae family) have a high fatty acids concentration (oleic, stearic and palmitic) and have demonstrated an important anti-oxidant activity [22,23] has pointed out that the administration of an antioxidant therapy may significantly reduce the oxidative effects in patients that have been treated with benznidazole and suggests that the combination of both (benznidazole treatment and the antioxidant therapy) is capable of reduce oxidative damage in the heart and other organs affected by *T. cruzi*.

Carvahalo *et al.* [24] also described important pathological changes registered in rats with E vitamin deficiency, and it was observed along the sub-acute phase of *T. cruzi* infection a lot of pathological lesions including an increased myocarditis, sympatric denervation, leucopoenia and a major monocites to macrophages differentiation.

Several studies have reported that the crude extract of *C. papaya* also has shown good activity against gastrointestinal nematodes such as *Trichostrongylus colubriformis* [25], *Ascaris suum* in pigs [26], *Haemoncus contortus* in sheep [27] and *Trichuris muris* in mice [28] attributing the activity of the crude extract of *C. papaya* against gastrointestinal nematodes because the presence of cysteine protease enzymes in the extract.

It must be considered that by increasing the inflammatory process, the compromise and the cardiac injury degree rises, therefore the administration of this crude extract made from *C. papaya* seeds could be promising due to its direct activity against the causal agent, and in the modification of the antioxidant levels (fatty acids), which produce a favorable effect in the treated individual, especially during the initial stage of the disease.

## 4. Experimental

### 4.1. Parasites and Animals

Trypomastigotes of *T. cruzi* strain H4 were used. Eight weeks old BALB/c mice were maintained on a 12:12 h light-dark cycle and had access to food and water *ad libitum.* To evaluate the acute phase of American tripanosomiasis each mouse was IP-inoculated with 1 × 10^3^ *T. cruzi* trypomastigotes. 

### 4.2. Plant Material and Preparation of Extract

A honey dew variety of papaya seed (*C. papaya* L.) was collected from Escarcega Campeche, Mexico for use in this study. Fresh seeds from ripe fruits were shade-dried for 15 days and later coarsely powdered. In a 1 L bottom flask fitted with a reflux condenser, 100 g of dry ground papaya seed and 500 mL of chloroform were heated for 4 h, cooled to room temperature and filtered. The solvent was dried under a vacuum in a rotary evaporator and then dried in a vacuum oven at room temperature for 12 h [18].

### 4.3. *In Vivo* Antiprotozoal Activity against T. cruzi during Acute Phase of the Disease

For the evaluation of the acute phase of American Trypanosomiasis *in vivo*, two different bioassays were conducted. In each bioassay, BALB/c mice were randomly divided into six groups (*n* = 6 each). Groups were infected with 1 × 10^3^ trypomastigotes through intraperitoneal injection (IP): a positive control group, a negative control group and other four groups of mice for the evaluation of the different experimental doses.

For the first bioassay, two different doses of the crude chloroform extract were evaluated: 50 and 75 mg/kg. Similarly, a compound mixture including oleic, palmitic and stearic acids from the chloroform extract was evaluated by administering it orally (in a concentration of 100 mg/kg). The proportion of each compound was calculated as a function of the percentage that each one of them usually in the crude chloroform extracts of *C. papaya* [12]*.* Immediately before administration, the crude chloroform extract was mixed with phosphate buffered saline (PBS, 137 mM NaCl, 2.7 mM KCl, 4.3 mM Na_2_HPO_4_, and 1.4 mM KH_2_PO_4_, pH 7.4), and administered orally every 24 h for 28 days. In the second bioassay, only the compound mixtures were evaluated, but at doses of 200 and 300 mg/kg.

For each bioassay control groups were considered. As negative control, a group of infected mice received only 50 µL of the vehicle (PBS) orally, whilst a positive control group including infected mice was treated orally with allopurinol (8.5 mg/kg) diluted in 50 µL of PBS every day for the entire duration of the study.

To estimate the parasitemia degree in both bioassays, mice were examined (blood trypomastigotes counts) every 4 days for 28 days after the inoculation. Treated animals were compared with the control group. The days when each mouse died was also recorded to estimate the mortality rate for each group. 

To determine *C. papaya* activity against the intracellular amastigote form of *T. cruzi*, cardiac tissue samples from treated and untreated mice were collected and fixed in formaldehyde (10%). Afterwards, the paraffin-embedded tissue sections were stained with Hematoxylin-Eosin (HE) and examined under a light microscope. Four non-consecutive slides from the heart of each mouse were also examined in a blinded mode. The number of amastigote nests was quantified in 100 zones for each heart. All procedures were conducted in accordance with the internationally accepted principles for laboratory animal use and care [29].

### 4.4. Statistical Analysis

Data are expressed as means ± S.E.M. and statistical analyses were performed using the Student’s t-test (*p* < 0.05), and ANOVA followed by Tukey’s multiple comparison test was used to compare more than two groups. 

## 5. Conclusions

In this study we have demonstrated that the chloroform extract of *C. papaya* has significant antiprotozoal activity, but does not totally eliminate *T. cruzi* trypomastigotes during the active phase of infection. However, this is a promising potential drug to use associated with other commonly used anti-*T. cruzi* drugs, such as benznidazol, for improving therapeutic effectiveness.
